# Phenotyping immune sensitization reveals pattern-based association with post-lung transplant complications

**DOI:** 10.1016/j.jhlto.2026.100639

**Published:** 2026-07-17

**Authors:** Mark John Siringan, Taisuke Kaiho, Rachel Reichenbach, Ambalavanan Arunachalam, Yudai Miyashita, Yuriko Yagi, Ankit Bharat, Chitaru Kurihara

**Affiliations:** a Division of Thoracic Surgery, Department of Surgery, Northwestern University Feinberg School of Medicine, Chicago, IL; b Division of Pulmonary and Critical Care Medicine, Department of Medicine, Northwestern University Feinberg School of Medicine, Chicago, IL

**Keywords:** Lung transplantation, Phenotyping, Sensitization, FEV1, CLAD, Survival

## Abstract

Immune sensitization is an important contributor to adverse outcomes after lung transplantation, including impaired pulmonary function, increased risk of chronic lung allograft dysfunction (CLAD), and reduced survival. Prior studies have examined individual immunologic features, such as panel reactive antibodies (PRA), donor-specific antibodies (DSA), acute cellular rejection (ACR), and antibody-mediated rejection (AMR). However, existing literature has evaluated each of these in isolation, creating a gap in understanding regarding the potential for synergistic and overlapping effects. In this retrospective cohort study, we developed 5 immune sensitization phenotypes based on perioperative patterns of immune injury observed before and after transplantation: non-sensitized, pre-transplant humoral sensitization, post-transplant humoral sensitization, cellular-mediated sensitization, and mixed sensitization. A total of 502 adult lung transplant recipients were classified into these phenotypes and compared with respect to initial post-transplant FEV_1_, CLAD risk, and overall survival. Across outcomes, the mixed sensitization phenotype was generally associated with worse post-transplant lung function and greater mortality, while CLAD onset was directionally similar but remained statistically nonsignificant. Within the mixed sensitization group, the AMR subtype demonstrated significant associations with adverse lung outcomes. These findings suggest that immune sensitization may be better understood as overlapping patterns of immune injury that develop over time after transplant. These findings provide an exploratory framework for describing longitudinal immune injury patterns that may guide future studies of post-transplant surveillance and enable tailored immunomodulatory strategies.

## Background

Lung transplantation remains the definitive treatment for end-stage lung disease; however, long-term outcomes remain limited, with a median post-transplant survival of approximately 6 years.^1^, ^2^ This limited survival is in contrast to other solid organ transplants, including kidney, liver, and pancreas, which have nearly double the median survival.^3^ One of the most important contributors to graft injury following transplantation is immune sensitization.4., 5 Sensitization may occur pre-transplant, manifesting as elevated panel reactive antibodies (PRA), pre-transplant donor-specific antibodies (DSA), or historical DSA; or post-transplant, through the development of de novo DSA, acute cellular rejection (ACR), or antibody-mediated rejection (AMR).

Individual immunologic markers of sensitization have been extensively studied in relation to post-transplant outcomes. Elevated PRA has been associated with increased post-operative ventilatory burden and a higher risk of chronic lung allograft dysfunction (CLAD), particularly bronchiolitis obliterans syndrome (BOS).^6^, ^7^, ^8^ Prior studies have also demonstrated that both pre-transplant and de novo DSA are associated with accelerated FEV_1_ decline, increased risk of baseline lung allograft dysfunction, and reduced survival.^9^ Patients with pre-transplant DSA were also found to have greater post-operative ventilatory burden, and patients with de novo DSA were found to have an increased risk for CLAD.^10^, ^11^, ^12^, ^13^ Similarly, both ACR and AMR events have been independently linked to increased CLAD risk and reduced survival.^14^, ^15^

While these studies consistently demonstrate that immune sensitization impacts lung transplant outcomes, these immunologic features are frequently examined in isolation. In clinical practice, however, lung transplant recipients often exhibit a spectrum of combined immune sensitization patterns. Evaluating immunologic markers individually may not fully capture how multiple forms of immune injury coexist and evolve over time in clinical practice.

To address this gap in existing literature, we consolidated multiple perioperative immunologic features, including PRA, DSA, ACR, and AMR, into sensitization phenotypes and evaluated their associations with post-transplant lung function, CLAD, and survival outcomes. We hypothesized that different sensitization phenotypes would demonstrate distinct longitudinal outcome patterns after transplantation.

## Materials and methods

### Study design

**Figure 1:**
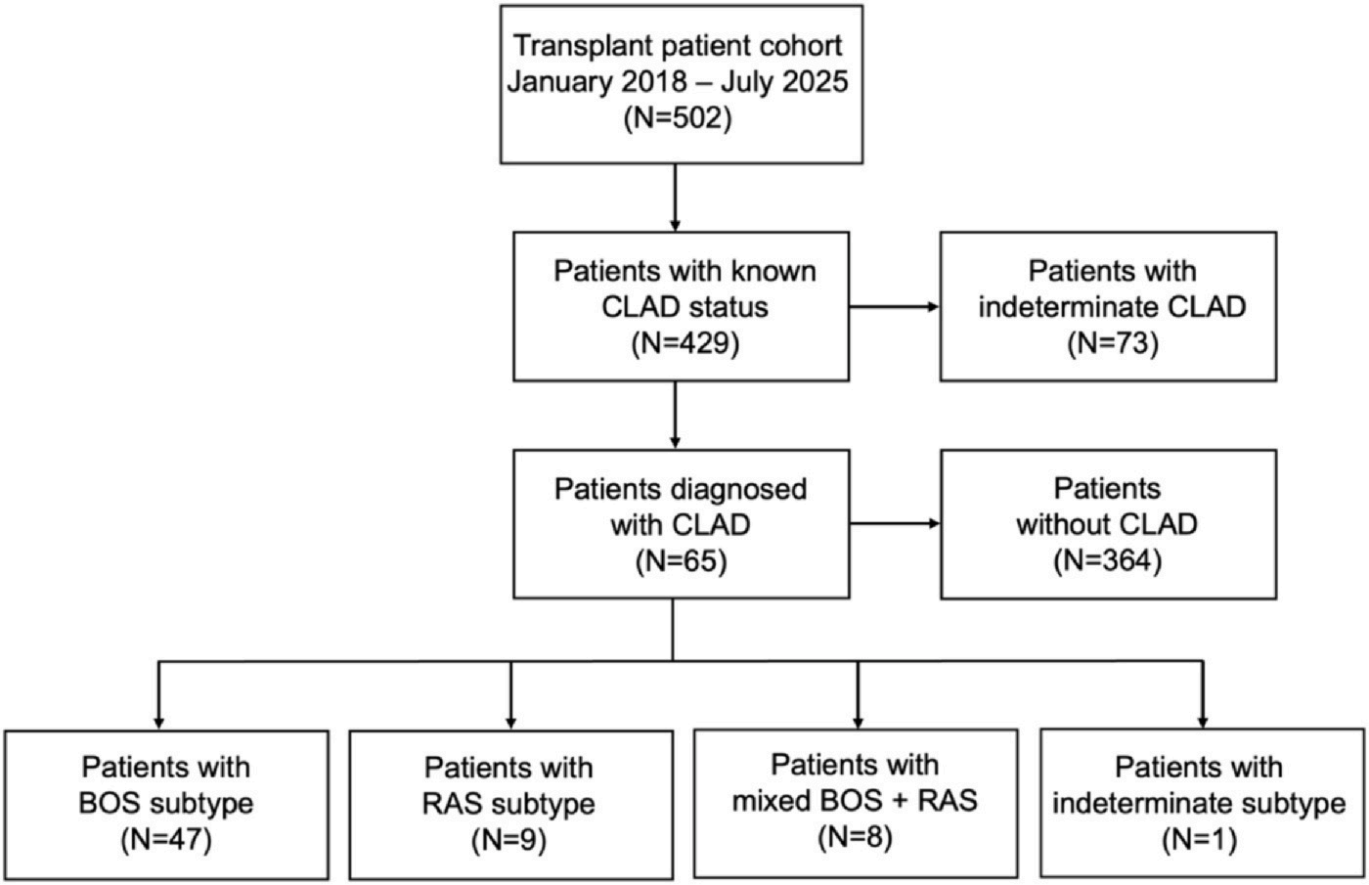
Flowchart of patient cohort, stratified by FEV_1_ and CLAD outcomes. FEV_1_, forced expiratory reserve volume in 1 second; CLAD, chronic lung allograft dysfunction; BOS, bronchiolitis obliterans syndrome; RAS, restrictive allograft syndrome.

This is a retrospective cohort study of 502 adult patients who underwent lung transplantation at a single institution between January 2018 and July 2025 (Figure 1). Patient data were collected using electronic medical records and stored in a secure database at the Northwestern University Medical Center in Chicago, Illinois, USA. The study was approved by the Institutional Review Board of Northwestern University (STU00212120). All lung transplants in this study were performed at Northwestern Memorial Hospital in accordance with institutional protocols, United States regulations, the Declaration of Helsinki, and the principles of the Declaration of Istanbul on Organ Trafficking and Transplant Tourism; no organs were procured from prisoners or other unethical sources. Our institutional lung transplant peri- and post-operative management has been described previously.^16^

### Definition of immunological sensitization phenotypes

Five immunological sensitization phenotypes were defined using clinically recognized immune complications occurring before and after transplantation.^15^, ^17^, ^18^ The phenotypes were built using perioperative immune features, including PRA assay percentage, historic DSA, pre-transplant DSA, de novo DSA, recurrent ACR, and AMR (Table 1). For PRA, the maximum of either PRA class I or class II was used to determine phenotype classification. A PRA threshold of 25% was selected to distinguish low- and high-risk sensitization, consistent with prior studies identifying this cutoff as prognostically meaningful.^8^ Post-transplant immune events, including de novo DSA, recurrent ACR, and AMR, were tracked across the available follow-up period after transplantation. The median follow-up duration of the study cohort was 1.71 years (IQR 0.96-3.27 years). Using these features, the following 5 phenotypes were created: non-sensitized, pre-transplant humoral sensitization, post-transplant humoral sensitization, cellular-mediated sensitization, and mixed sensitization. The mixed sensitization group was then further subdivided into an ACR-type and an AMR-type. ACR and AMR history are combined as they reflect a shared pattern of injury involving both humoral and cellular-mediated sensitization.

**Table 1:** Definitions of Immunologic Sensitization Phenotypes

Immunologic variable	Non-sensitized	Pre-transplant humoral	Post-transplant humoral	Cellular-mediated	Mixed	
					AMR type	ACR type
PRA ≥ 25%	—	⊕	○	—	○	⊕
Historic DSA	—	⊕	○	—	○	⊕
Pre-transplant DSA	—	⊕	○	—	○	⊕
De novo DSA	—	—	●	—	○	⊕
Recurrent ACR (≥ 2 events)	—	—	—	●	—	●
AMR	—	—	—	—	●	—

— Absent; ○ Allowed to be present; ⊕ One or more must be present; ● Required.

The non-sensitized group lacks features of pre- or post-transplant sensitization. Pre-transplant humoral sensitization is defined by antibody-mediated markers present before transplantation. Post-transplant humoral sensitization is characterized by the development of donor-specific antibodies without recurrent cellular rejection. Cellular-mediated sensitization is defined by recurrent T-cell–mediated rejection. Mixed sensitization is defined as overlapping humoral and cellular immune mechanisms, characterized by antibody-mediated rejection or by recurrent cellular rejection with concurrent humoral sensitization. PRA refers to the maximum of either PRA class I or class II. PRA cutoff of 25% chosen for its reported significance in lung transplant prognosis.^8^ ACR, acute cellular rejection; AMR, antibody-mediated rejection; DSA, donor-specific antibody; PRA, panel reactive antibody.

Because several of these immune events occur after transplantation, these groups were not intended to represent fixed risk categories present at the time of transplant. Instead, they were created to describe the different patterns of immune injury patients experienced over time after transplantation.

For this work, the term “phenotype” is used to describe clinically defined patterns of immune injury observed over time after transplantation rather than biologically intrinsic or data-derived phenotypes.

Patients were assigned to each phenotype based on their constellation of immune features through a hierarchical approach to ensure each patient was attributed to only 1 phenotype (Figure S1). First, patients with a post-transplant AMR event, without history of ACR, were assigned AMR-type mixed sensitization, and patients with recurrent (≥ 2) ACR events with concurrent pre- or post-transplant humoral sensitization were assigned ACR-type mixed sensitization. Next, patients with no AMR or ACR but with de novo DSA were assigned post-transplant humoral sensitization. Then, patients with only recurrent ACR and none of the other immune features were assigned cellular-mediated sensitization. Afterwards, patients with one or more of pre-transplant DSA, historical DSA, or PRA ≥ 25% were assigned pre-transplant humoral sensitization. Lastly, patients lacking all selected immune features were assigned to the non-sensitized group.

### Definition of outcomes

The outcomes of this study were FEV_1_ trajectory post-transplant, CLAD, and overall survival. CLAD is defined according to the 2019 International Society of Heart and Lung Transplantation consensus report: substantial and persistent decline (≥ 20%) in measured FEV_1_ from the baseline value in the absence of other etiologies, where baseline is defined as the average of the highest 2 post-operative FEV_1_ measurements taken at least 3 weeks apart.^19^

### Statistical analysis

Analyses of FEV_1_ were restricted to patients with at least 2 post-transplant FEV_1_ measurements. CLAD analyses included only patients with known CLAD status, while survival analyses included all 502 transplant recipients.

Continuous data are presented as median (Interquartile range), and discrete data are presented as numbers (%). Baseline recipient characteristics, peri-operative clinical features, and immunological sensitization markers were compared between phenotypes (Table 2). The Kruskal-Wallis test was used to compare continuous data, while Chi-square and Fisher’s exact tests were used to compare categorical data.

**Table 2:** Characteristics of Patients and Perioperative Course

Characteristic	Non-sensitized (*N* = 293)	Pre-transplant humoral (*N* = 46)	Post-transplant humoral (*N* = 51)	Cellular mediated (*N* = 57)	Mixed (*N* = 55)	*P* value
Age (year)	63 [56-68]	56 [49-64]	57 [47-66]	64 [56-69]	62 [54-68]	0.001^a^
Sex						
Female	103 (35.2%)	37 (80.4%)	28 (54.9%)	21 (36.8%)	27 (49.1%)	<0.001^b^
Male	190 (64.8%)	9 (19.6%)	23 (45.1%)	36 (63.2%)	28 (50.9%)	<0.001^b^
Race						
White	203 (69.3%)	20 (43.5%)	31 (60.8%)	43 (75.4%)	36 (65.5%)	0.005^b^
Black	32 (10.9%)	13 (28.3%)	8 (15.7%)	5 (8.8%)	11 (20.0%)	0.010^b^
Hispanic	30 (10.2%)	10 (21.7%)	6 (11.8%)	5 (8.8%)	4 (7.3%)	0.152^b^
Asian	14 (4.8%)	2 (4.3%)	3 (5.9%)	3 (5.3%)	3 (5.5%)	0.978^c^
Other	14 (4.8%)	1 (2.2%)	3 (5.9%)	1 (1.8%)	1 (1.8%)	0.716^c^
BMI (kg/m²)	26.3 [21.9-29.2]	26.7 [23.6-29.3]	26.6 [22.6-30.7]	26.3 [22.4-30.3]	26.6 [23.0-29.7]	0.854^a^
Smoking history	159 (54.3%)	19 (41.3%)	24 (47.1%)	33 (57.9%)	27 (49.1%)	0.382^b^
Etiology						
ILD	144 (49.1%)	19 (41.3%)	23 (45.1%)	30 (52.6%)	27 (49.1%)	0.803^b^
COPD	60 (20.5%)	6 (13.0%)	4 (7.8%)	14 (24.6%)	12 (21.8%)	0.136^b^
PAH	18 (6.1%)	6 (13.0%)	7 (13.7%)	5 (8.8%)	3 (5.5%)	0.188^c^
ARDS	16 (5.5%)	9 (19.6%)	7 (13.7%)	2 (3.5%)	4 (7.3%)	0.007^c^
Other	55 (18.8%)	6 (13.0%)	10 (19.6%)	6 (10.5%)	9 (16.4%)	0.543^b^
Donor age (y)	35 [25-46]	38 [28-48]	36 [28-46]	29 [21–39]	37 [28-44]	0.196^a^
ECMO use	184 (62.8%)	38 (82.6%)	35 (68.6%)	26 (45.6 %)	33 (60.0%)	0.003^b^
Surgery type						
SOLT	106 (36.2%)	9 (19.6%)	12 (23.5%)	25 (43.9%)	26 (47.3%)	0.009^b^
BOLT	187 (63.8%)	37 (80.4%)	39 (76.5%)	32 (56.1%)	29 (52.7%)	0.009^b^
Ischemic time (hour)	5.5 [4.4-7.1]	5.6 [5.2-6.7]	5.6 [4.7-6.3]	4.9 [3.9-5.6]	5.2 [3.7-6.3]	<0.001^a^
OR time (hour)	5.1 [3.8-6.4]	6.8 [4.6-8.1]	5.9 [4.8-7.7]	5.7 [4.9-7.5]	5.5 [3.9-7.3]	<0.001^a^
Intra-op transfusion						
pRBC	0 [0-2]	2 [0-7]	2 [0-5]	0 [0-1]	0 [0-2]	<0.001^a^
FFP	0 [0-0]	0 [0-2]	0 [0-2]	0 [0-0]	0 [0-0]	0.009^a^
PLT	0 [0-0]	0 [0-2]	0 [0-1]	0 [0-0]	0 [0-0]	<0.001^a^
Post-op						
PGD grade 3	40 (13.7%)	10 (21.7%)	9 (17.6%)	5 (8.8%)	8 (14.5%)	0.398^b^
CLAD	28 (9.6%)	9 (19.6%)	4 (7.8%)	13 (22.8%)	11 (20%)	0.008^b^
ICU stay (d)	6 [4-14]	10 [6-21]	10 [4-17]	6 [5–10]	9 [5-24]	0.004^a^
Vent days (d)	2 [1-3]	3 [1-8]	2 [1-4]	1 [1–3]	2 [1-5]	<0.001^a^
Deaths	58 (19.8%)	13 (28.3%)	6 (11.8%)	19 (33.3%)	24 (43.6%)	<0.001^b^
PRA (%)						
Class I	0.0 [0.0-0.0]	38.5 [8.5-54.8]	8.0 [0.0-47.0]	0.0 [0.0-0.0]	7.0 [0.0-54.0]	<0.001^a^
Class II	0.0 [0.0-0.0]	0.0 [0.0-31.5]	0.0 [0.0-22.5]	0.0 [0.0-0.0]	0.0 [0.0-7.5]	<0.001^a^
ACR episodes	0 [0-0]	0 [0-0]	0 [0-0]	3 [2-4]	2 [0-2]	<0.001^a^
AMR episodes	0 [0-0]	0 [0-0]	0 [0-0]	0 [0-0]	1 [0-2]	<0.001^a^
Historic DSA	0 (0.0%)	6 (13.0%)	8 (15.7%)	0 (0.0%)	5 (9.1%)	<0.001^c^
Pre-tx DSA	0 (0.0%)	28 (60.9%)	22 (43.1%)	0 (0.0%)	24 (43.6%)	<0.001^b^
De novo DSA	0 (0.0%)	0 (0.0%)	51 (100.0%)	0 (0.0%)	31 (56.4%)	<0.001^b^
Retro XM T-cell	1 (0.3%)	12 (26.1%)	17 (33.3%)	0 (0.0%)	9 (16.4%)	<0.001^c^
Retro XM B-cell	2 (0.7%)	14 (30.4%)	17 (33.3%)	0 (0.0%)	12 (21.8%)	<0.001^c^

Continuous data are shown as median [interquartile range], and discrete data are shown as number (%). ACR, acute cellular rejection; AMR, antibody-mediated rejection; ARDS, acute respiratory distress syndrome; BMI, body mass index; BOLT, bilateral orthotopic lung transplantation; COPD, chronic obstructive pulmonary disease; DSA, donor-specific antibody; ECMO, extracorporeal membrane oxygen; FFP, fresh frozen plasma; ILD, interstitial lung disease; PAH, pulmonary arterial hypertension; PGD, primary graft dysfunction; PLT, platelet; PRA, panel reactive antibody; pRBC, packed red blood cells; Pre-tx, pre-transplant; Retro XM, retrospective crossmatch; SOLT, single orthotopic lung transplantation. ECMO use includes any ECMO use before, during, and after lung transplantation. ^a^Kruskal-Wallis, ^b^Chi-square, ^c^Fisher’s exact test.

Kaplan-Meier curves were constructed for each phenotype, depicting time to ≥ 20% decline from peak FEV_1_, CLAD, and death. Pairwise log-rank comparisons between Kaplan-Meier curves were conducted to directly compare phenotypes against 1 another. Restricted mean survival time (RMST) was calculated for each phenotype relative to the non-sensitized group at post-transplant years 1 through 5. A stacked distribution plot was constructed for FEV_1_ outcomes, illustrating the proportion of patients with ≥ 20% FEV_1_ decline, preserved FEV_1_, or censoring at each time point.

Since it is possible for immune exposures, such as ACR or AMR, to occur after FEV_1_ decline or CLAD onset, we performed a patient-level assessment comparing the timing of ACR and AMR relative to FEV_1_ decline and CLAD onset (Figures S2 and S3). Patients included in this analysis were restricted to those with known CLAD or FEV_1_ decline status and available timing data for CLAD, ACR, and AMR. De novo DSA timing was not available and was therefore not included in our time sequence assessment.

For post-transplant FEV_1_ baseline analyses, univariable associations were evaluated using linear mixed-effects regression models incorporating time since transplant and a random intercept to account for repeated FEV_1_ measurements within patients. CLAD risk was assessed using univariable logistic regression, and overall survival was analyzed using univariable Cox proportional hazards models. Multivariable models were subsequently constructed, adjusting for variables that were either statistically significant in univariable analyses or have been previously reported contributors to post-transplant lung function.^20^, ^21^, 22. All multivariable models included adjustment for sensitization phenotype, age, sex, transplant type, transplant etiology, pre-operative PaO_2_ and hemoglobin, donor age, ischemic time, operating time, peri-operative ECMO use, and primary graft dysfunction (PGD) grade 3. The phenotype variable incorporated the immune features used to define the sensitization groups. The multivariable linear mixed-effects model for post-transplant FEV_1_ additionally included a time-since-transplant term and a phenotype x time interaction term to model FEV_1_ trajectory. Subgroup analyses comparing AMR-type versus ACR-type mixed sensitization included an AMR variable with ACR as the reference. For FEV_1_ analysis within the mixed sensitization subgroup, the full covariate set was retained.

Due to limited event counts, CLAD subtype models were only adjusted for age, while the survival subtype models were adjusted for age, PGD grade 3, and operating time.

To further validate our findings with respect to the temporal relationship between immune exposures and post-transplant outcomes, a 6-month landmark sensitivity analysis was performed, with follow-up initiated 6 months post-transplant and phenotypes reassigned using only pre-landmark immune information. Patients with de novo DSA were excluded due to unavailable timing data, and ACR classification was based on the timing of the first event due to a lack of longitudinal event timing. Survival analyses included only patients alive at the landmark, and CLAD-free survival analyses included only patients alive and free of CLAD at the landmark. Kaplan-Meier curves were calculated to reassess outcomes under this landmark analysis. FEV_1_ analyses were not conducted due to low event rates following restriction to post-landmark follow-up.

Statistical significance was set at *p* < 0.05.

All statistical analyses were performed using R Version 4.4.3.

## Results

### Patient characteristics

The study cohort included 502 lung transplant recipients. Patients in the cellular-mediated group had an older median age, whereas those in the pre-transplant humoral group were generally younger (Table 2). The cohort was predominantly Caucasian across all phenotypes. There was a higher proportion of females among pre- and post-transplant humoral sensitization patients, while the other phenotypes were predominantly male. Smoking history did not differ significantly across phenotypes. Acute respiratory distress syndrome (ARDS) etiology was the most prevalent etiology of lung failure prior to transplant in the pre-transplant humoral sensitization group and the least prevalent in the cellular-mediated group. Perioperative ECMO use was also highest among patients with pre-transplant humoral sensitization and lowest in the cellular-mediated group. Bilateral lung transplantation was more common than single lung transplantation across all phenotypes. Median ischemic time and operating time were longest in the pre-transplant humoral sensitization group. This group also received higher intra-operative transfusion volumes across all blood products, including packed red blood cells, fresh frozen plasma, and platelets (PLT). Retrospective B- and T-cell crossmatch positivity was most frequently observed in the post-transplant humoral sensitization group.

### Post-transplant clinical outcomes

PGD grade 3 did not differ significantly across sensitization phenotypes (Table 2). In contrast, CLAD prevalence varied by phenotype, with the highest prevalence observed in the cellular-mediated group and the lowest in the post-transplant humoral group. Patients with pre-transplant humoral sensitization had longer median durations of post-operative ICU stay and mechanical ventilation. Overall mortality differed across phenotypes, with the highest proportion of deaths occurring in mixed sensitization patients.

In patient-level lead-time plots, ACR and AMR events consistently preceded both FEV_1_ decline and CLAD onset in phenotypes defined by post-transplant events, supporting a consistent temporal sequence (Figures S2 and S3).

### Post-transplant FEV1 across sensitization phenotypes

**Figure 2:**
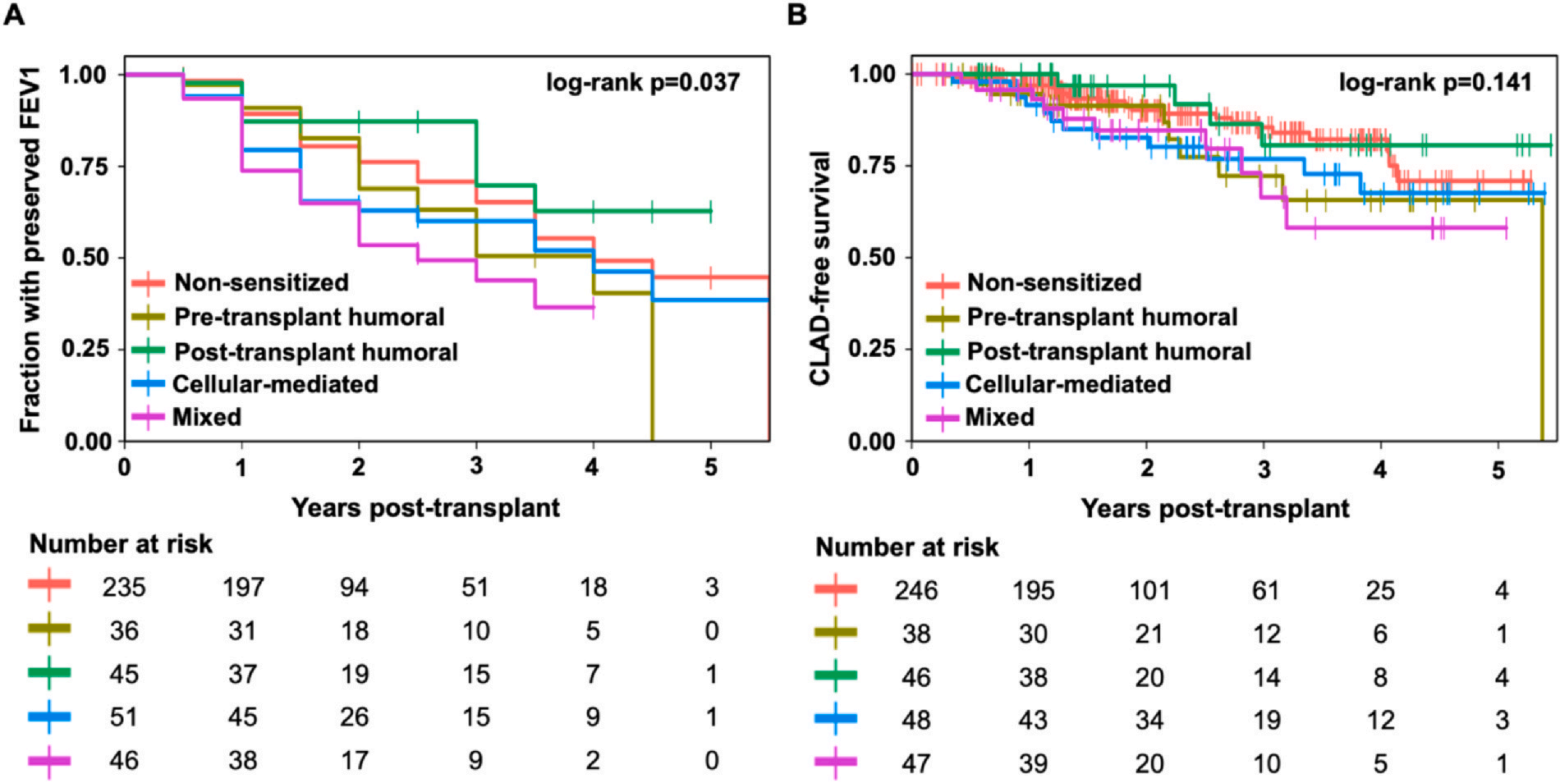
Kaplan-Meier curves of lung function endpoints, showing time to at least 20% decline in FEV_1_ from the patient’s post-transplant peak and CLAD-free survival, stratified by immunological phenotype. The tables summarize the number of patients at risk for each time point. (**A**) Curves depict the fraction of patients remaining that have not yet experienced at least a 20% decline from peak FEV_1_. The global log-rank *p*-value of 0.037 indicates a significant difference in lung function decline across phenotypes. Only patients with at least 2 post-transplant FEV_1_ measurements were included in this analysis. (**B**) Curves depict the fraction of patients who are still alive and have not developed CLAD. The global log-rank *p*-value of 0.141 indicates no significant difference in CLAD-free survival between phenotypes. Only patients with known CLAD status are included in this analysis.

Kaplan-Meier curves, depicting time to ≥ 20% decline in FEV_1_ from an individual’s post-transplant peak, showed a significant difference across phenotypes (Figure 2A, log-rank *p* = 0.037). Pairwise log-rank comparisons showed the mixed phenotype had earlier FEV1 decline compared to non-sensitized patients, though it was not statistically significant (Table S1, *p* = 0.078). A stacked distribution plot similarly highlighted a consistently higher proportion of FEV_1_ decline in the mixed sensitization group compared with other phenotypes (Figure S4).

Univariable linear mixed effects models were constructed to evaluate associations with post-transplant baseline FEV_1_ (Table S2). Among immunologic variables, higher PRA reactivity, pre-transplant DSA, de novo DSA, positive retrospective B- and T-cell crossmatching, and AMR events were associated with lower baseline FEV_1_. Desensitization therapy and plasmapheresis were also associated with reduced lung function. Higher pre-operative hemoglobin levels were associated with slightly higher FEV_1_. Older donor age and female donor sex were associated with slightly lower lung function.

A multivariable linear mixed-effects model was subsequently constructed, following adjustments as outlined in the methods section. The analysis showed a significant interaction between time and post-transplant humoral sensitization, which indicated that, despite lower baseline FEV_1_, this group demonstrated greater relative recovery of lung function over time compared with non-sensitized patients (Table 3, β = 0.10, *p* < 0.001). The post-transplant humoral sensitization group demonstrated a lower post-transplant FEV_1_ compared to non-sensitized patients, though this result was non-significant (β = −0.17, *p* = 0.078). Similarly, the mixed sensitization group had reduced FEV_1_ compared to non-sensitized patients, consistent with the previously described trend in the Kaplan-Meier curve analysis (β = −0.19, *p* = 0.053).

**Table 3:** Multivariable Linear Mixed-Effects Regression for Post-Transplant FEV_1_ Baseline and Trajectory

Variable	Estimate (β)	95% CI	*p*-value
Time after transplant (years)	−0.01	−0.03, 0.01	0.467
Phenotype			
Pre-transplant humoral sensitization	−0.06	−0.28, 0.15	0.558
Post-transplant humoral sensitization	−0.17	−0.36, 0.02	0.078
Cellular-mediated sensitization	−0.13	−0.32, 0.05	0.166
Mixed sensitization	−0.19	−0.39, 0.00	0.053
Age (year)	−0.00	−0.01, 0.01	0.959
Female	−0.45	−0.57, −0.33	<0.001
BOLT	0.36	0.18, 0.53	<0.001
Etiology			
ILD	0.03	−0.12, 0.18	0.706
PAH	−0.03	−0.25, 0.20	0.826
ARDS	−0.14	−0.40, 0.13	0.312
Hemoglobin (g/dL)	0.01	−0.01, 0.04	0.244
PaO₂ (mmHg)	0.00	0.00, 0.00	0.005
Donor age (year)	−0.01	−0.01, 0.00	<0.001
PGD grade 3	−0.10	−0.29, 0.09	0.325
Ischemic time (hour)	0.00	−0.02, 0.02	0.987
OR time (hour)	−0.01	−0.05, 0.03	0.687
ECMO use	−0.15	−0.29, 0.00	0.055
Interaction terms			
Time × Pre-transplant humoral sensitization	−0.01	−0.06, 0.04	0.731
Time × Post-transplant humoral sensitization	0.10	0.06, 0.15	<0.001
Time × Cellular-mediated sensitization	0.02	−0.02, 0.05	0.314
Time × Mixed sensitization	0.04	−0.02, 0.09	0.161

The −0.19 estimate for the “mixed sensitization” variable depicts a suggestive trend for low lung function early after transplant compared to the non-sensitized group (*p* = 0.053). Interaction terms are included to study time-dependent changes in lung function across phenotypes. The 0.10 estimate for the “time x post-transplant sensitization” variable is significant and indicates FEV_1_ recovery over time (*p* < 0.001). Adjusted β estimates for FEV_1_ baseline and trajectory are included in the table. ARDS, acute respiratory distress syndrome; BOLT, bilateral orthotopic lung transplantation; COPD, chronic obstructive pulmonary disease; ECMO, extracorporeal membrane oxygenation; ILD, interstitial lung disease; PAH, pulmonary arterial hypertension; PGD, primary graft dysfunction. Intercept represents modeled baseline lung function, measured by FEV_1_. ECMO use includes any ECMO use before, during, and after lung transplantation. Patients with at least 2 post-transplant FEV_1_ measurements were included in this analysis.

### CLAD development across sensitization phenotypes

Kaplan–Meier curves, depicting time to CLAD, suggested earlier CLAD onset in the mixed and pre-transplant humoral groups, although differences were not statistically significant (Figure 2B, log-rank *p* = 0.141). Additionally, in a multivariable Cox proportional hazards model for CLAD-free survival, mixed sensitization patients demonstrated a directional, though nonsignificant, trend towards shorter CLAD-free survival compared with non-sensitized patients (Table S3, HR = 2.15, *p* = 0.059). RMST analyses were performed to compare phenotypic differences in CLAD-free survival relative to the non-sensitized group (Table S4). Although no significant differences were found, there is a numerically reduced CLAD-free survival by 4.6 months in the mixed sensitization phenotype compared with the non-sensitized phenotype at 4 years post-transplant (RMST difference = −0.38 years, *p* = 0.064).

Univariable logistic regression models were constructed to evaluate associations with CLAD risk (Table S5). A history of ACR events was associated with increased risk of developing CLAD. Additionally, longer operating time was associated with a greater risk of CLAD, while longer ischemic time showed a slight reduction in CLAD risk.

A multivariable logistic regression model was subsequently constructed, following adjustments as outlined in the methods section (Table 4). In accordance with previous trends, the mixed sensitization group demonstrated numerically higher odds of CLAD compared to the non-sensitized group, although this association did not reach statistical significance (OR = 2.36, *p* = 0.060).

**Table 4:** Multivariable Logistic Regression for CLAD Risk

Variable	OR	95% CI	*p*-value
Phenotype			
Pre-transplant humoral sensitization	1.85	0.67, 4.82	0.216
Post-transplant humoral sensitization	0.52	0.14, 1.59	0.292
Cellular-mediated sensitization	1.28	0.53, 2.96	0.567
Mixed sensitization	2.36	0.94, 5.70	0.060
Recipient factors			
Age (year)	0.99	0.96, 1.01	0.325
Female	0.89	0.45, 1.73	0.736
BOLT	0.46	0.18, 1.19	0.111
Etiology			
ILD	0.78	0.35, 1.79	0.539
PAH	0.68	0.19, 2.16	0.521
ARDS	0.56	0.13, 2.18	0.412
Hemoglobin (g/dL)	1.12	0.99, 1.29	0.086
PaO₂ (mmHg)	1.00	1.00, 1.00	0.197
Donor age (year)	0.99	0.97, 1.02	0.619
PGD grade 3	0.59	0.18, 1.63	0.346
Ischemic time (hour)	0.88	0.73, 1.01	0.118
OR time (hour)	1.71	1.38, 2.13	<0.001
ECMO use	0.49	0.22, 1.10	0.084

There is no significant association between the 4 phenotypes and CLAD risk; however, the *p*-value of 0.060 and odds ratio (OR) of 2.36 for “mixed sensitization” are aligned with the general trend of a mixed phenotype being associated with worsened lung outcomes compared to the non-sensitized group. Adjusted odds ratios for CLAD-free survival are depicted in the table. ARDS, acute respiratory distress syndrome; BOLT, bilateral orthotopic lung transplantation; COPD, chronic obstructive pulmonary disease; ECMO, extracorporeal membrane oxygenation; ILD, interstitial lung disease; PAH, pulmonary arterial hypertension; PGD, primary graft dysfunction. ECMO use includes any ECMO use before, during, and after lung transplantation. Only patients with known CLAD status are included in this analysis.

### Overall survival across sensitization phenotypes

**Figure 3:**
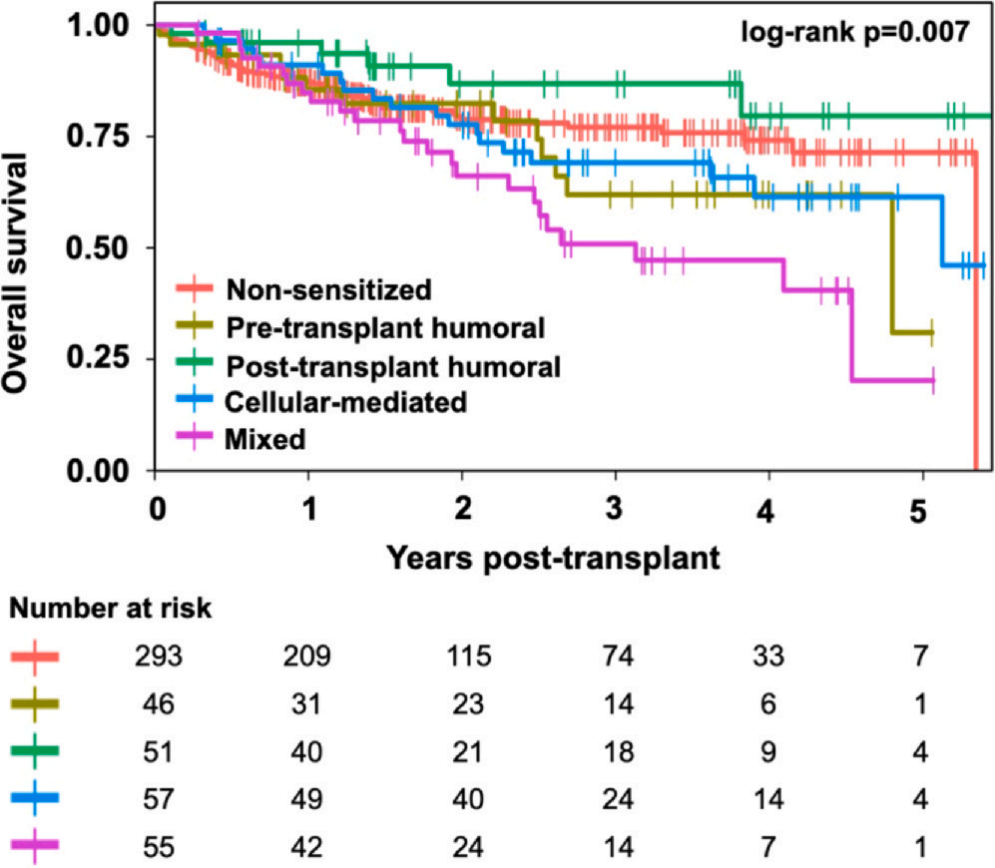
Kaplan-Meier curves for overall survival across different phenotypes. Curves depict the fraction of patients still alive. The global log-rank *p*-value of 0.007 indicates a significant difference in the survival curves between phenotypes. The table summarizes the number of patients at risk for each time point. All 502 patients were included in this analysis.

Kaplan-Meier curves, depicting overall survival, demonstrated a significant difference across sensitization phenotypes (Figure 3, log-rank *p* = 0.007). Pairwise comparisons demonstrated significantly reduced survival in the mixed sensitization phenotype compared with the non-sensitized (Table S6, *p* = 0.022) and post-transplant humoral sensitization groups (*p* = 0.016). RMST analyses supported these findings, showing reduced survival for the mixed sensitization phenotype compared to non-sensitized at 4 and 5 years post-transplant, corresponding to an approximate 6 (*p* = 0.017) and eleven-month (*p* = 0.001) reduction in survival time, respectively (Table S7).

Univariable Cox proportional hazards models were constructed to evaluate associations with overall survival (Table S8). A history of chronic kidney disease was associated with worse survival, whereas bilateral lung transplantation was associated with a lower risk of mortality. Post-operative plasmapheresis was associated with an increased risk of mortality. Among laboratory variables, higher creatinine and total bilirubin levels were modestly associated with increased risk. PGD grade 3 was also associated with an increased mortality risk.

A multivariable Cox proportional hazards model was constructed following adjustments outlined in the methods section (Table 5). Similar to previous trends, the mixed sensitization group exhibited a significantly higher risk of mortality compared to non-sensitized patients (Figure S5, HR = 1.85, *p* = 0.016).

**Table 5:** Multivariable Cox Proportional Hazards Model for Overall Survival

Variable	HR	95% CI	*p*-value
Phenotype			
Pre-transplant humoral	1.40	0.73, 2.68	0.315
Post-transplant humoral	0.60	0.25, 1.42	0.243
Cellular-mediated	1.28	0.72, 2.25	0.400
Mixed	1.85	1.12, 3.06	0.016
Recipient factors			
Age	1.01	0.99, 1.03	0.528
Female	1.21	0.81, 1.80	0.361
BOLT	0.43	0.24, 0.78	0.005
Etiology			
ILD	1.05	0.60, 1.85	0.859
PAH	0.46	0.16, 1.29	0.139
ARDS	0.81	0.33, 1.98	0.648
Hemoglobin	1.01	0.94, 1.09	0.793
PaO2	1.00	1.00, 1.00	0.763
Donor age	1.01	1.00, 1.03	0.165
PGD grade 3	2.73	1.74, 4.28	<0.001
Ischemic time (h)	0.97	0.90, 1.05	0.410
OR time (h)	1.11	0.98, 1.26	0.106
ECMO	1.49	0.89, 2.50	0.133

The hazard ratio (HR) of 1.85 for the “Mixed” sensitization indicates that the mixed group is associated with a greater risk of mortality compared to the non-sensitized group (*p* = 0.016). Adjusted hazard ratios for overall survival are depicted in the table. ARDS, acute respiratory distress syndrome; BOLT, bilateral orthotopic lung transplantation; COPD, chronic obstructive pulmonary disease; ECMO, extracorporeal membrane oxygenation; ILD, interstitial lung disease; PAH, pulmonary arterial hypertension; PGD, primary graft dysfunction. ECMO use includes any ECMO use before, during, and after lung transplantation. All 502 patients were included in this analysis.

### Mixed sensitization AMR-type vs. ACR-type sub-analysis

A subgroup analysis was performed within the mixed sensitization phenotype to evaluate whether AMR or ACR-subtypes differentially contributed to the associations observed between mixed sensitization and worsened outcomes.

In a multivariable linear mixed-effects model, the AMR-subtype was associated with significantly lower baseline FEV_1_ compared with the ACR-subtype (Table S9, β = −0.422, *p* = 0.022). A truncated multivariable logistic regression model additionally demonstrated no significant difference in CLAD risk between AMR- and ACR- subtypes, although the AMR-subtype demonstrated higher odds of CLAD (Table S10, OR = 2.34, *p* = 0.232). Finally, in a multivariable Cox proportional hazards model, the AMR-subtype demonstrated a borderline association with increased mortality risk relative to the ACR-subtype (Table S11, HR = 2.17, *p* = 0.078).

### Landmark sensitivity analysis

Table S12 outlines the number of patients in each phenotype after imposing a 6-month post-transplant landmark, which was broadly similar to the patient distribution in the main analysis. In this landmark analysis, overall survival remained significantly associated with phenotypes, showing greater mortality in the mixed phenotype and demonstrating a similar pattern of immune injury to the main analysis (Figure S6). Associations with CLAD-free survival were attenuated and not statistically significant (Figure S7). FEV_1_ decline was not assessed due to low post-landmark event rates.

## Discussion

In this study, we defined 5 immunological sensitization phenotypes representing different patterns of immune complications before and after lung transplantation. These phenotypes were created to describe longitudinal immune courses rather than fixed biologic risk categories. We uncovered 2 key findings: (1) the mixed sensitization phenotype was consistently associated with reduced lung function, a trend toward increased CLAD risk, and higher mortality, and (2) within the mixed phenotype, the AMR-subtype had a stronger association with worsened pulmonary outcomes than the ACR-subtype. Notably, a landmark sensitivity analysis demonstrated similar post-transplant outcomes.

Notably, pre-transplant humoral, post-transplant humoral, and cellular-mediated sensitization did not differ significantly from non-sensitized patients with respect to FEV_1_, CLAD, or survival. This finding is consistent with prior reports showing nonsignificant differences between certain allosensitized and non-sensitized patients.^23^, ^24^, ^25^, ^26^ Our findings highlight the heterogeneity of immune sensitization after lung transplantation. While DSA or a high PRA are often viewed as relative contraindications to transplantation at many centers, our results suggest that sensitization should be interpreted within its broader clinical context.^27^, ^28^, ^29^, ^30^ Our previously reported desensitization protocol may partially explain why several sensitization phenotypes did not demonstrate significantly worse outcomes in this cohort.^16^ If validated, longitudinal sensitization patterns may provide a complementary framework for describing post-transplant immune courses.

Limitations of this study include its retrospective, single-institution nature. Additionally, limited event counts restricted power in subgroup analyses, including comparisons between AMR- and ACR-subtypes, necessitating truncated multivariable models for CLAD and survival outcomes. While our approach incorporated several immune complications, additional dimensions of rejection severity, such as PRA percentile stratification, were not evaluated.^31^ baseline lung allograft dysfunction was not systematically captured, and insufficient post-transplant FEV_1_, CLAD, and de novo DSA data limited further subgroup analyses.^32^, ^33^ Restriction of FEV_1_ and CLAD analyses to patients with sufficient follow-up may have introduced selection bias, particularly among patients with early mortality. Because several immune events used to define these phenotypes occurred after transplantation, analyses anchored at transplant remain susceptible to immortal time bias. Although the landmark sensitivity analysis demonstrated broadly similar findings, it only partially addressed this limitation because de novo DSA timing was unavailable. Furthermore, this exploratory study was not designed to determine whether the phenotype-based framework provides greater predictive value than individual immune markers, which warrants direct comparison in future studies. Accordingly, these phenotypes should be interpreted as descriptive clinical groupings rather than biologically derived phenotypes, and these findings remain exploratory and hypothesis-generating.

## Conclusion

Our findings suggest that considering overlapping immune complications accrued over time may provide a useful descriptive framework for studying post-transplant sensitization. Future multi-institutional studies comparing this framework directly with individual immune markers are needed to determine its potential clinical utility.

## Conflicts of Interest statement

The authors declare the following financial interests/personal relationships, which may be considered as potential competing interests: Chitaru Kurihara reports financial support was provided by NIH. If there are other authors, they declare that they have no known competing financial interests or personal relationships that could have appeared to influence the work reported in this paper.

## Footnotes

Appendix A Supplementary data associated with this article can be found in the online version at doi:10.1016/j.jhlto.2026.100639.
