# Supplementary material for: Phenotyping immune sensitization reveals pattern-based association with post-lung transplant complications
Source: JHLT Open. 2026 Jul 17;14:100639. doi: 10.1016/j.jhlto.2026.100639 (PMC13473965; doi:10.1016/j.jhlto.2026.100639)
Supplement: Supplementary file 1 [file mmc1.docx]

**Phenotyping Immune Sensitization Reveals Pattern-Based Association with Post-Lung Transplant Complications**

Mark John Siringan BA^1^, Taisuke Kaiho MD^1^, Rachel Reichenbach MD^1^, Ambalavanan Arunachalam MD^2^, Yudai Miyashita MD^1^, Yuriko Yagi MD^1^, Ankit Bharat MD^1^, Chitaru Kurihara MD^1^

^1^Division of Thoracic Surgery, Department of Surgery, Northwestern University Feinberg School of Medicine, Chicago, Illinois, USA.

^2^Division of Pulmonary and Critical Care Medicine, Department of Medicine, Northwestern University Feinberg School of Medicine, Chicago, Illinois, USA

**Supplementary Material**

**Table of Contents**

Supplementary methods………………………………………………………………………...3-4

Figure S1. Schematic of hierarchical phenotype assignment……………………………………..5

Figure S2. Time-lead plot for time sequence to FEV_1_ decline………………………………….6-7

Figure S3. Time-lead plot for time sequence to CLAD onset…………………………………….8

Figure S4. Stacked distribution plot for FEV_1_ outcomes………………………………………….9

Figure S5. Forest plot for overall survival ……………………………………………..………..10

Figure S6. Kaplan-Meier curve for overall survival in 6-month landmark analysis………...…..11

Figure S7. Kaplan-Meier curve for CLAD-free survival in 6-month landmark analysis…….….12

Table S1. Pairwise log-rank comparisons between FEV_1_ Kaplan Meier curves………………...13

Table S2. Univariable linear mixed-effects regression for baseline FEV_1_..………………….14-16

Table S3. Multivariable Cox proportional hazards model for CLAD-free survival…………17-18

Table S4. Restricted mean survival time for CLAD-free survival.………………..……...….19-20

Table S5. Univariable logistic regression for CLAD development ……..…………………...21-23

Table S6. Pairwise log-rank comparisons between overall survival Kaplan Meier curves...........24

Table S7. Restricted mean survival time for overall survival……………………………......25-26

Table S8. Univariable Cox proportional hazards model for overall survival ………...……...27-29

Table S9. Multivariable linear mixed-effects regression for AMR vs. ACR……………...... 30-31

Table S10. Multivariable logistic regression for AMR vs. ACR………...……….………..…….32

Table S11. Multivariable Cox proportional hazards model for AMR vs. ACR ………..……….33

Table S12. Patient counts in phenotypes for 6-month landmark analysis……………..………...34

**Supplementary Methods**

*Statistical Analysis*

Pairwise log-rank comparisons between Kaplan-Meier curves were conducted to directly compare phenotypes against one another. Restricted mean survival time (RMST) was calculated for each phenotype relative to the non-sensitized group at post-transplant years 1 through 5. A stacked distribution plot was constructed for FEV_1_ outcomes, illustrating the proportion of patients with ≥20% FEV_1_ decline, preserved FEV_1_, or censoring at each time point.

Since it is possible for immune exposures, such as ACR or AMR, to occur after FEV_1_ decline or CLAD onset, we performed a patient-level assessment comparing the timing of ACR and AMR relative to FEV_1_ decline and CLAD onset. Patients included in this analysis were restricted to those with known CLAD or FEV_1_ decline status and available timing data for CLAD, ACR, and AMR. De novo DSA timing was not available and was therefore not included in our time sequence assessment.

For post-transplant FEV_1_ baseline analyses, univariable associations were evaluated using linear mixed-effects regression models incorporating time since transplant and a random intercept to account for repeated FEV_1_measurements within patients. CLAD risk was assessed using univariable logistic regression, and overall survival was analyzed using univariable Cox proportional hazards models. Multivariable models were subsequently constructed, adjusting for variables that were either statistically significant in univariable analyses or deemed clinically relevant to post-transplant lung function and survival. All multivariable models included adjustment for sensitization phenotype, age, sex, transplant type, transplant etiology, pre-operative PaO_2_ and hemoglobin, donor age, ischemic time, operating time, peri-operative ECMO use, and primary graft dysfunction (PGD) grade 3. The multivariable linear mixed-effects model for post-transplant FEV_1_ baseline and trajectory additionally included time since transplant and phenotype x time interaction terms to model changes in slope over time. Subgroup analyses comparing AMR-type versus ACR-type mixed sensitization included an AMR variable with ACR as the reference. For FEV_1_ trajectory within the mixed sensitization subgroup, the full covariate set was retained.

Due to limited event counts, CLAD subtype models were only adjusted for age, and survival subtype models were adjusted for age, PGD grade 3, and operating time.

Analyses of FEV₁ trajectory were restricted to patients with at least two post-transplant FEV_1_ measurements. CLAD analyses included only patients with known CLAD status, while survival analyses included all 502 transplant recipients.

To further validate our findings with respect to the temporal relationship between immune exposures and post-transplant outcomes, a 6-month landmark sensitivity analysis was performed, with follow-up initiated 6 months post-transplant and phenotypes reassigned using only pre-landmark immune information. Patients with de novo DSA were excluded due to unavailable timing data, and ACR classification was based on the timing of the first event due to a lack of longitudinal event timing. Survival analyses included only patients alive at the landmark, and CLAD-free survival analyses included only patients alive and free of CLAD at the landmark. Kaplan-Meier curves were calculated to reassess outcomes under this landmark analysis. FEV_1_ analyses were not conducted due to low event rates following restriction to post-landmark follow-up.

Statistical significance was set at p < 0.05. All statistical analyses were performed using R Version 4.4.3.

**Figure Legends**

**Figure S1.** Schematic depicting the hierarchical assignment of patients into their respective sensitization phenotypes.

**Figure S2**. Lead-time plot illustrating the time sequence of ACR, AMR, and ≥ 20% decline in FEV_1_ from post-transplant peak. The cohort includes patients from post-transplant sensitization phenotypes (cellular-mediated and mixed) who experienced an FEV_1_ decline event. Each line represents an individual patient, with ACR (blue), AMR (orange), FEV_1_ decline (red), and last follow-up (cross). Patients 1-22 correspond to the cellular-mediated phenotype, and patients 23-42 correspond to the mixed phenotype. Four patients (IDs 6, 33, 36, and 38) had documented FEV_1_ decline and immune events but lacked available timing for ACR/AMR and are therefore shown without corresponding immune event markers. Among all evaluable patients with known timing, immune events consistently preceded FEV_1_ decline.

**Figure S3**. Lead-time plot showing the time sequence of ACR, AMR, CLAD, death, and last follow-up. The cohort includes patients from post-transplant sensitization phenotypes (cellular-mediated and mixed). Each line represents an individual patient, with ACR (blue), AMR (orange), CLAD (red), death (cross), and last follow-up (circle). Patients 1-12 correspond to the cellular-mediated phenotype, and patients 13-22 correspond to the mixed phenotype. Three patients (IDs 18, 19, and 22) had documented CLAD and immune events but lacked available timing for ACR/AMR and are therefore shown without corresponding immune event markers. Among all evaluable patients with known timing, immune events consistently preceded CLAD onset.

**Figure S4**. Stacked distributions plot, depicting the proportion of patients over time without a 20% decline in FEV_1_ from personal peak (green), patients with a 20% decline in FEV_1_ from personal peak (red), and patients who were censored (grey). (**A**) Non-sensitized, (**B**) Pre-transplant humoral sensitization, (**C**) Post-transplant humoral sensitization, (**D**) Cellular-mediated sensitization, (**E**) Mixed sensitization.

**Figure S5**. Forest plot highlighting adjusted hazard ratios for overall survival of the four sensitized phenotypes relative to the non-sensitized phenotype. Confidence intervals that cross the vertical dashed line represent no significant difference in survival with the non-sensitized phenotype. The center dots represent the hazard ratio, and arms represent the width of the 95% confidence interval. Of the 4 phenotypes, the mixed sensitization group shows a significantly higher risk of death. Hazard ratios calculated from a multivariable Cox proportional hazards model (see Table 5.)

**Figure S6**. Kaplan-Meier curve for overall survival across modified phenotypes for landmark sensitivity analysis. This analysis uses 6 months post-transplant as the re-defined time-zero point from which follow-up is initiated. Curves depict the fraction of patients still alive. The global log-rank p-value of 0.027 indicates a significant difference in the survival curves between phenotypes. The table summarizes the number of patients at risk for each time point.

**Figure S7**. Kaplan-Meier curves of CLAD-free survival across modified phenotypes for landmark sensitivity analysis. This analysis uses 6 months post-transplant as the re-defined time-zero point from which follow-up is initiated. Curves depict the fraction of patients still at risk for CLAD, and CLAD-free survival was assessed from the landmark onwards. The global p-value of 0.595 indicates no significant difference.

**Table S1**. Pairwise log-rank comparisons of Kaplan-Meier curves for preserved FEV_1_.

| Comparison | χ² | Adjusted p-value |
| --- | --- | --- |
| Non-sensitized vs Pre-transplant humoral | 0.93 | 1.000 |
| Non-sensitized vs Post-transplant humoral | 0.95 | 1.000 |
| Non-sensitized vs Cellular-mediated | 1.37 | 1.000 |
| Non-sensitized **vs Mixed** | **7.08** | **0.078** |
| Pre-transplant humoral vs Post-transplant humoral | 2.61 | 0.745 |
| Pre-transplant humoral vs Cellular-mediated | 0.00 | 1.000 |
| Pre-transplant humoral vs Mixed | 1.65 | 1.000 |
| Post-transplant humoral vs Cellular-mediated | 3.18 | 0.595 |
| Post-transplant humoral **vs Mixed** | **6.07** | **0.124** |
| Cellular-mediated vs Mixed | 0.86 | 1.000 |

P-values were adjusted for multiple comparisons through Holm-correction. Despite a significant global log-rank p-value, no significant pairwise comparison remains after Holm-correction. The p-value of 0.078 for the comparison between non-sensitized and mixed phenotypes reflects the trend of a mixed phenotype being associated with worsened lung function. Only patients with at least two post-transplant FEV_1_ measurements were included in this analysis.

**Table S2**. Univariable linear mixed-effects regression for post-transplant baseline FEV_1_.

| Characteristics | Estimate (β) | 95% CI | p-value |
| --- | --- | --- | --- |
| Recipient factors |  |  |  |
| Age (y) | 0.00 | −0.01, 0.00 | 0.589 |
| Female | −0.50 | −0.61, −0.38 | **<0.001** |
| BMI (kg/m²) | 0.00 | −0.02, 0.01 | 0.579 |
| BSA (m²) | 0.82 | 0.59, 1.06 | **<0.001** |
| Smoking history | −0.01 | −0.13, 0.11 | 0.847 |
| Hypertension | −0.06 | −0.18, 0.07 | 0.371 |
| Diabetes | −0.09 | −0.22, 0.05 | 0.197 |
| CKD | −0.13 | −0.36, 0.11 | 0.288 |
| BOLT | 0.21 | 0.09, 0.34 | **0.001** |
| PRA | −0.23 | −0.36, −0.11 | **<0.001** |
| Pre-transplant DSA | −0.20 | −0.37, −0.02 | **0.027** |
| Historic DSA | −0.12 | −0.49, 0.24 | 0.507 |
| De novo DSA | −0.18 | −0.34, −0.02 | **0.025** |
| Desensitization | −0.34 | −0.53, −0.14 | **<0.001** |
| Pre-op plasmapheresis | −0.33 | −0.51, −0.14 | **<0.001** |
| Post-op plasmapheresis | −0.40 | −0.54, −0.26 | **<0.001** |
| Retro XM T-cell | −0.26 | −0.49, −0.04 | **0.023** |
| Retro XM B-cell | −0.28 | −0.50, −0.06 | **0.011** |
| ACR episodes | −0.02 | −0.06, 0.03 | 0.454 |
| AMR episodes | −0.15 | −0.29, −0.02 | **0.029** |
| Etiology |  |  |  |
| ILD | 0.03 | −0.14, 0.20 | 0.755 |
| COPD | −0.07 | −0.28, 0.13 | 0.488 |
| PAH | −0.06 | −0.31, 0.19 | 0.643 |
| ARDS | −0.07 | −0.34, 0.19 | 0.580 |
| Laboratory |  |  |  |
| Hemoglobin (g/dL) | 0.04 | 0.01, 0.06 | **0.002** |
| WBC (10³/mm³) | −0.01 | −0.02, 0.01 | 0.244 |
| Platelets (10³/mm³) | 0.00 | 0.00, 0.00 | 0.263 |
| Sodium (mEq/L) | −0.02 | −0.03, 0.00 | 0.091 |
| BUN (mg/dL) | 0.00 | 0.00, 0.01 | 0.298 |
| Creatinine (mg/dL) | 0.10 | −0.01, 0.21 | 0.083 |
| ALT (U/L) | 0.00 | 0.00, 0.01 | 0.080 |
| AST (U/L) | 0.00 | 0.00, 0.00 | 0.907 |
| Albumin (g/dL) | −0.01 | −0.03, 0.01 | 0.383 |
| Total Bilirubin (mg/dL) | 0.09 | −0.02, 0.20 | 0.096 |
| INR | 0.25 | −0.13, 0.63 | 0.191 |
| Arterial blood gas |  |  |  |
| pH | −0.52 | −1.40, 0.37 | 0.254 |
| PaCO₂ (mmHg) | 0.00 | 0.00, 0.01 | 0.288 |
| PaO₂ (mmHg) | 0.00 | 0.00, 0.00 | **0.015** |
| Donor |  |  |  |
| Donor age (y) | −0.01 | −0.01, −0.01 | **<0.001** |
| Donor female | −0.15 | −0.28, −0.03 | 0.019 |
| Intra-operative |  |  |  |
| OR time (h) | 0.01 | −0.02, 0.04 | 0.376 |
| pRBC transfusion | −0.01 | −0.03, 0.00 | 0.149 |
| FFP transfusion | 0.00 | −0.03, 0.02 | 0.759 |
| PLT transfusion | −0.01 | −0.06, 0.05 | 0.854 |
| Ischemic time (h) | 0.01 | −0.01, 0.02 | 0.511 |
| Post-operative |  |  |  |
| PGD grade 3 | −0.14 | −0.34, 0.07 | 0.187 |
| ECMO use | 0.01 | −0.12, 0.13 | 0.909 |

BMI, body mass index; BSA, body surface area; CKD, chronic kidney disease; BOLT, bilateral orthotopic lung transplantation; PRA, panel reactive antibody; DSA, donor-specific antibody; Retro XM, retrospective crossmatch; ACR, acute cellular rejection; AMR, antibody-mediated rejection; ILD, interstitial lung disease; COPD, chronic obstructive pulmonary disease; PAH, pulmonary arterial hypertension; ARDS, acute respiratory distress syndrome; WBC, white blood cell; BUN, blood urea nitrogen; ALT, alanine aminotransferase; AST, aspartate aminotransferase; INR, international normalized ratio; pRBC, packed red blood cells; FFP, fresh frozen plasma; PLT, platelet; PGD, primary graft dysfunction; ECMO, extracorporeal membrane oxygenation. ECMO use includes any ECMO use before, during, and after lung transplantation. Only patients with at least two post-transplant FEV_1_ measurements were included in this analysis.

**Table S3**. Multivariable Cox proportional hazards model for CLAD-free survival.

| Variable | HR | 95% CI | p-value |
| --- | --- | --- | --- |
| **Phenotype** |  |  |  |
| Pre-transplant humoral sensitization | 2.05 | 0.86, 4.88 | 0.106 |
| Post-transplant **humoral** sensitization | 0.53 | 0.17, 1.62 | 0.266 |
| Cellular-mediated sensitization | 1.24 | 0.58, 2.67 | 0.581 |
| Mixed sensitization | 2.15 | 0.97, 4.77 | 0.059 |
| **Recipient factors** |  |  |  |
| Age (y) | 0.98 | 0.96, 1.01 | 0.180 |
| Female | 0.79 | 0.42, 1.47 | 0.453 |
| BOLT | 0.77 | 0.31, 1.89 | 0.566 |
| **Etiology** |  |  |  |
| ILD | 0.74 | 0.35, 1.58 | 0.437 |
| PAH | 0.53 | 0.17, 1.63 | 0.271 |
| ARDS | 0.65 | 0.20, 2.10 | 0.470 |
| Hemoglobin (g/dL) | 1.15 | 1.01, 1.31 | 0.036 |
| PaO₂ (mmHg) | 1.00 | 1.00, 1.00 | 0.581 |
| Donor age (y) | 1.00 | 0.98, 1.02 | 0.908 |
| PGD grade 3 | 0.80 | 0.30, 2.13 | 0.661 |
| Ischemic time (h) | 0.89 | 0.75, 1.06 | 0.197 |
| OR time (h) | 1.27 | 1.04, 1.55 | 0.018 |
| ECMO use | 0.81 | 0.38, 1.73 | 0.581 |

There is no significant association between the four phenotypes and CLAD-free survival; however, the p-value of 0.059 and hazard ratio (HR) of 2.15 for “mixed sensitization” are aligned with the general trend of a mixed phenotype being associated with worsened lung outcomes compared to the non-sensitized group. Adjusted hazard ratios for CLAD-free survival are depicted in the table. BOLT, bilateral orthotopic lung transplantation; ILD, interstitial lung disease; COPD, chronic obstructive pulmonary disease; PAH, pulmonary arterial hypertension; ARDS, acute respiratory distress syndrome; PGD, primary graft dysfunction; ECMO, extracorporeal membrane oxygenation. ECMO use includes any ECMO use before, during, and after lung transplantation. Only patients with known CLAD status are included in this analysis.

**Table S4**. Restricted mean survival time (RMST) differences for CLAD-free survival.

| Years post-transplant | Comparison to non-sensitized group | RMST difference (years) | 95% CI | p-value |
| --- | --- | --- | --- | --- |
| 1 | Pre-transplant humoral | −0.01 | −0.04, 0.02 | 0.475 |
|  | Post-transplant humoral | **+0.01** | 0.00, 0.02 | **0.022** |
|  | Cellular-mediated | −0.01 | −0.04, 0.02 | 0.517 |
|  | Mixed | −0.01 | −0.04, 0.02 | 0.450 |
| 2 | Pre-transplant humoral | −0.03 | −0.15, 0.09 | 0.620 |
|  | Post-transplant humoral | +0.05 | −0.01, 0.11 | 0.094 |
|  | Cellular-mediated | −0.09 | −0.21, 0.02 | 0.122 |
|  | Mixed | −0.07 | −0.19, 0.05 | 0.232 |
| 3 | Pre-transplant humoral | −0.13 | −0.37, 0.11 | 0.284 |
|  | Post-transplant humoral | +0.07 | −0.08, 0.22 | 0.376 |
|  | Cellular-mediated | −0.19 | −0.42, 0.04 | 0.108 |
|  | Mixed | −0.15 | −0.39, 0.09 | 0.218 |
| 4 | Pre-transplant humoral | −0.29 | −0.71, 0.12 | 0.162 |
|  | Post-transplant humoral | +0.05 | −0.27, 0.36 | 0.775 |
|  | Cellular-mediated | −0.29 | −0.65, 0.08 | 0.121 |
|  | Mixed | **−0.38** | −0.79, 0.02 | **0.064** |
| 5 | Pre-transplant humoral | −0.36 | −0.98, 0.26 | 0.261 |
|  | Post-transplant humoral | +0.13 | −0.37, 0.64 | 0.606 |
|  | Cellular-mediated | −0.33 | −0.86, 0.19 | 0.215 |
|  | Mixed | −0.52 | −1.15, 0.11 | 0.107 |

The +0.01 RMST difference for the comparison between post-transplant humoral and non-sensitized groups suggests an associated lower development of CLAD in the sensitized group (p=0.022). However, this association decays with time. The p-value of 0.064 and RMST difference of -0.38 for the comparison between mixed to non-sensitized groups is aligned with the general trend that a mixed phenotype is associated with worsened lung function. No other statistically significant results are noted. Only patients with known CLAD status are included in this analysis.

**Table S5**. Univariable logistic regression for CLAD risk.

| Variable | OR | 95% CI | p-value |
| --- | --- | --- | --- |
| Recipient factors |  |  |  |
| Age (y) | 0.98 | 0.96, 1.00 | 0.058 |
| Female | 0.86 | 0.50, 1.47 | 0.581 |
| BMI (kg/m²) | 1.01 | 0.95, 1.07 | 0.738 |
| BSA (m²) | 0.90 | 0.31, 2.62 | 0.843 |
| Smoking history | 1.02 | 0.60, 1.72 | 0.954 |
| Hypertension | 0.63 | 0.37, 1.07 | 0.085 |
| Diabetes | 1.23 | 0.70, 2.15 | 0.472 |
| CKD | 0.96 | 0.36, 2.59 | 0.940 |
| BOLT | 1.03 | 0.59, 1.78 | 0.925 |
| PRA | 1.12 | 0.66, 1.92 | 0.670 |
| Pre-transplant DSA | 1.07 | 0.51, 2.22 | 0.863 |
| Historic DSA^1^ | — | — | — |
| De novo DSA | 1.24 | 0.64, 2.42 | 0.525 |
| Desensitization | 0.31 | 0.09, 1.03 | 0.056 |
| Pre-op plasmapheresis | 0.67 | 0.27, 1.64 | 0.379 |
| Post-op plasmapheresis | 0.98 | 0.53, 1.84 | 0.962 |
| Retro XM T-cell | 0.54 | 0.16, 1.82 | 0.319 |
| Retro XM B-cell | 1.02 | 0.41, 2.54 | 0.966 |
| ACR episodes | 1.34 | 1.14, 1.57 | **<0.001** |
| AMR episodes | 1.88 | 0.88, 4.02 | 0.102 |
| **Etiology** |  |  |  |
| ILD | 0.75 | 0.38, 1.49 | 0.412 |
| COPD^1^ | — | — | — |
| PAH | 1.01 | 0.35, 2.92 | 0.992 |
| ARDS | 1.17 | 0.40, 3.44 | 0.771 |
| Laboratory |  |  |  |
| Hemoglobin (g/dL) | 1.01 | 0.91, 1.13 | 0.831 |
| WBC (10³/mm³) | 1.02 | 0.96, 1.09 | 0.495 |
| Platelets (10³/mm³) | 1.00 | 1.00, 1.00 | 0.793 |
| Sodium (mEq/L) | 1.00 | 0.93, 1.08 | 0.972 |
| BUN (mg/dL) | 1.00 | 0.97, 1.03 | 0.961 |
| Creatinine (mg/dL) | 0.51 | 0.17, 1.53 | 0.227 |
| ALT (U/L) | 1.00 | 0.98, 1.01 | 0.606 |
| AST (U/L) | 1.00 | 0.99, 1.02 | 0.532 |
| Albumin (g/dL) | 0.98 | 0.85, 1.13 | 0.774 |
| Total Bilirubin (mg/dL) | 1.25 | 0.83, 1.89 | 0.290 |
| INR | 2.73 | 0.62, 11.95 | 0.183 |
| Arterial blood gas |  |  |  |
| pH | 0.04 | 0.00, 1.97 | 0.106 |
| PaCO₂ (mmHg) | 1.00 | 0.98, 1.02 | 0.743 |
| PaO₂ (mmHg) | 1.00 | 1.00, 1.00 | 0.267 |
| Donor age (y) | 0.99 | 0.97, 1.01 | 0.161 |
| Donor female | 0.79 | 0.44, 1.41 | 0.422 |
| Intra-operative |  |  |  |
| OR time (h) | 1.31 | 1.15, 1.50 | **<0.001** |
| pRBC transfusion | 1.04 | 0.97, 1.12 | 0.247 |
| FFP transfusion | 1.10 | 0.98, 1.24 | 0.108 |
| PLT transfusion | 1.09 | 0.87, 1.38 | 0.456 |
| Ischemic time (h) | 0.87 | 0.78, 0.98 | **0.024** |
| Post-operative |  |  |  |
| PGD grade 3 | 0.71 | 0.27, 1.89 | 0.499 |
| ECMO use | 0.91 | 0.53, 1.55 | 0.718 |

BMI, body mass index; BSA, body surface area; CKD, chronic kidney disease; BOLT, bilateral orthotopic lung transplantation; PRA, panel reactive antibody; DSA, donor-specific antibody; Retro XM, retrospective crossmatch; ACR, acute cellular rejection; AMR, antibody-mediated rejection; ILD, interstitial lung disease; COPD, chronic obstructive pulmonary disease; PAH, pulmonary arterial hypertension; ARDS, acute respiratory distress syndrome; WBC, white blood cell; BUN, blood urea nitrogen; ALT, alanine aminotransferase; AST, aspartate aminotransferase; INR, international normalized ratio; OR, operating room; pRBC, packed red blood cells; FFP, fresh frozen plasma; PLT, platelet; PGD, primary graft dysfunction; ECMO, extracorporeal membrane oxygenation. ECMO use includes any ECMO use before, during, and after lung transplantation. Only patients with known CLAD status are included in this analysis. ^1^Insufficient data to reliably calculate odds ratios.

**Table S6**. Pairwise log-rank comparisons of Kaplan-Meier curves for overall survival.

| Comparison | χ² | Adjusted p-value |
| --- | --- | --- |
| **Non-sensitized** vs Pre-transplant humoral | 1.09 | 0.891 |
| **Non-sensitized** vs Post-transplant humoral | 2.30 | 0.646 |
| **Non-sensitized** vs Cellular-mediated | 0.76 | 0.891 |
| **Non-sensitized vs Mixed** | **9.14** | **0.022** |
| Pre-transplant humoral vs Post-transplant humoral | 3.87 | 0.393 |
| Pre-transplant humoral vs Cellular-mediated | 0.13 | 0.891 |
| Pre-transplant humoral vs Mixed | 1.72 | 0.761 |
| Post-transplant humoral vs Cellular-mediated | 3.31 | 0.413 |
| **Post-transplant humoral vs Mixed** | **9.93** | **0.016** |
| Cellular-mediated vs Mixed | 3.70 | 0.393 |

P-values were adjusted for multiple comparisons through Holm correction. The p-value of 0.022 for the “non-sensitized vs mixed” sensitization comparison supports a significantly lower overall survival with a mixed phenotype compared to the non-sensitized phenotype. Similarly, the p-value of 0.016 for the post-transplant vs mixed sensitization comparison supports a significantly lower survival in a mixed phenotype. All 502 patients were included in this analysis.

**Table S7**. Restricted mean survival time (RMST) differences for overall survival.

| Year post-transplant | Comparison to non-sensitized group | RMST difference (years) | 95% CI | p value |
| --- | --- | --- | --- | --- |
| 1 | Pre-transplant humoral | 0.02 | −0.05, 0.08 | 0.639 |
|  | Post-transplant humoral | 0.05 | 0.00, 0.10 | 0.056 |
|  | Cellular-mediated | 0.04 | −0.01, 0.08 | 0.112 |
|  | Mixed | 0.03 | −0.02, 0.08 | 0.225 |
| 2 | Pre-transplant humoral | 0.02 | −0.16, 0.19 | 0.842 |
|  | Post-transplant humoral | **0.14** | **0.01, 0.26** | **0.033** |
|  | Cellular-mediated | 0.05 | −0.09, 0.18 | 0.500 |
|  | Mixed | −0.03 | −0.18, 0.12 | 0.700 |
| 3 | Pre-transplant humoral | −0.04 | −0.34, 0.26 | 0.798 |
|  | Post-transplant humoral | 0.23 | −0.01, 0.46 | 0.057 |
|  | Cellular-mediated | −0.02 | −0.27, 0.23 | 0.869 |
|  | Mixed | −0.22 | −0.50, 0.05 | 0.109 |
| 4 | Pre-transplant humoral | −0.18 | −0.63, 0.28 | 0.441 |
|  | Post-transplant humoral | 0.32 | −0.03, 0.67 | 0.073 |
|  | Cellular-mediated | −0.11 | −0.48, 0.27 | 0.580 |
|  | **Mixed** | **−0.51** | **−0.92, −0.09** | **0.017** |
| 5 | Pre-transplant humoral | −0.34 | −0.96, 0.28 | 0.284 |
|  | Post-transplant humoral | 0.40 | −0.10, 0.90 | 0.114 |
|  | Cellular-mediated | −0.21 | −0.73, 0.30 | 0.423 |
|  | **Mixed** | **−0.91** | **−1.46, −0.35** | **0.001** |

The RMST difference between mixed and non-sensitized groups was -0.51 years at 4 years post-transplant (p = 0.017) and worsened to -0.91 years at 5 years post-transplant (p = 0.001), indicating that a mixed phenotype promotes a progressively worsening survival over time and has a late divergence from the non-sensitized sensitization group in terms of overall survival. Post-transplant humoral sensitization demonstrates a significant association with improved survival compared to non-sensitized patients by 2 years post-transplant (p=0.033). However, this association decays with time. All 502 patients were included in this analysis.

**Table S8**. Univariable Cox proportional hazards for overall survival.

| Variable | HR | 95% CI | p-value |
| --- | --- | --- | --- |
| Recipient factors |  |  |  |
| Age (y) | 1.01 | 1.00, 1.03 | 0.114 |
| Female | 1.21 | 0.84, 1.73 | 0.302 |
| BMI (kg/m²) | 1.03 | 0.99, 1.07 | 0.176 |
| BSA (m²) | 1.15 | 0.55, 2.44 | 0.707 |
| Smoking history | 1.03 | 0.72, 1.48 | 0.862 |
| Hypertension | 1.10 | 0.76, 1.58 | 0.620 |
| Diabetes | 1.40 | 0.97, 2.03 | 0.073 |
| **CKD** | **2.05** | **1.22, 3.43** | **0.007** |
| **BOLT** | **0.59** | **0.41, 0.85** | **0.004** |
| PRA | 1.29 | 0.90, 1.84 | 0.171 |
| Pre-transplant DSA | 1.22 | 0.75, 1.99 | 0.432 |
| Historic DSA | 0.39 | 0.05, 2.83 | 0.355 |
| De novo DSA | 0.96 | 0.59, 1.56 | 0.874 |
| Desensitization | 1.00 | 0.50, 1.98 | 0.992 |
| Pre-op plasmapheresis | 1.23 | 0.70, 2.16 | 0.464 |
| **Post-op plasmapheresis** | **1.71** | **1.16, 2.52** | **0.007** |
| Retro XM T-cell | 0.72 | 0.29, 1.78 | 0.482 |
| Retro XM B-cell | 1.28 | 0.67, 2.46 | 0.451 |
| ACR episodes | 1.05 | 0.94, 1.18 | 0.389 |
| AMR episodes | 1.54 | 0.96, 2.47 | 0.072 |
| Etiology |  |  |  |
| ILD | 1.41 | 0.83, 2.40 | 0.208 |
| COPD | 5.84 | 0.77, 44.51 | 0.089 |
| PAH | 0.61 | 0.22, 1.66 | 0.331 |
| ARDS | 1.11 | 0.50, 2.46 | 0.790 |
| Laboratory |  |  |  |
| Hemoglobin (g/dL) | 0.99 | 0.92, 1.06 | 0.770 |
| WBC (10³/mm³) | 0.98 | 0.93, 1.02 | 0.329 |
| Platelets (10³/mm³) | 1.00 | 1.00, 1.00 | 0.840 |
| Sodium (mEq/L) | 1.00 | 0.95, 1.06 | 0.935 |
| BUN (mg/dL) | 1.00 | 0.98, 1.02 | 0.897 |
| **Creatinine (mg/dL)** | **1.02** | **1.01, 1.04** | **0.011** |
| ALT (U/L) | 1.00 | 0.99, 1.01 | 0.700 |
| AST (U/L) | 1.01 | 1.00, 1.01 | 0.085 |
| Albumin (g/dL) | 0.73 | 0.51, 1.03 | 0.074 |
| **Total Bilirubin (mg/dL)** | **1.31** | **1.05, 1.64** | **0.016** |
| INR | 1.22 | 0.45, 3.32 | 0.694 |
| Arterial blood gas |  |  |  |
| pH | 0.15 | 0.01, 1.90 | 0.143 |
| PaCO₂ (mmHg) | 0.99 | 0.98, 1.01 | 0.481 |
| PaO₂ (mmHg) | 1.00 | 1.00, 1.00 | 0.539 |
| Donor |  |  |  |
| Donor age (y) | 1.01 | 0.99, 1.02 | 0.262 |
| Donor female | 1.17 | 0.80, 1.70 | 0.413 |
| Intra-operative |  |  |  |
| OR time (h) | 0.98 | 0.90, 1.07 | 0.660 |
| pRBC transfusion | 1.00 | 0.96, 1.05 | 0.855 |
| FFP transfusion | 1.01 | 0.94, 1.09 | 0.724 |
| PLT transfusion | 1.03 | 0.89, 1.19 | 0.703 |
| Ischemic time (h) | 0.94 | 0.87, 1.02 | 0.116 |
| Post-operative |  |  |  |
| **PGD grade 3** | **2.63** | **1.72, 4.02** | **<0.001** |
| ECMO use | 0.96 | 0.66, 1.39 | 0.830 |

BMI, body mass index; BSA, body surface area; CKD, chronic kidney disease; BOLT, bilateral orthotopic lung transplantation; PRA, panel reactive antibody; DSA, donor-specific antibody; Retro XM, retrospective crossmatch; ACR, acute cellular rejection; AMR, antibody-mediated rejection; ILD, interstitial lung disease; COPD, chronic obstructive pulmonary disease; PAH, pulmonary arterial hypertension; ARDS, acute respiratory distress syndrome; WBC, white blood cell; BUN, blood urea nitrogen; ALT, alanine aminotransferase; AST, aspartate aminotransferase; INR, international normalized ratio; pRBC, packed red blood cells; FFP, fresh frozen plasma; PLT, platelet; PGD, primary graft dysfunction; ECMO, extracorporeal membrane oxygenation. ECMO use includes any ECMO use before, during, and after lung transplantation. All 502 patients were included in this analysis.

**Table S9**. Multivariable linear mixed-effects regression for FEV_1_ comparing ACR vs AMR-driven mixed sensitization.

| Variable | Estimate (β) | 95% CI | p-value |
| --- | --- | --- | --- |
| Time after transplant (y) | 0.023 | −0.066, 0.111 | 0.613 |
| **Mixed–AMR (reference: Mixed–ACR)** | **−0.422** | **−0.780, −0.064** | **0.022** |
| **Recipient factors** |  |  |  |
| Age (y) | 0.007 | −0.007, 0.022 | 0.328 |
| Female | −0.394 | −0.724, −0.064 | 0.020 |
| BOLT | 0.213 | −0.323, 0.750 | 0.428 |
| Etiology |  |  |  |
| ILD | −0.227 | −0.660, 0.206 | 0.297 |
| PAH | −0.729 | −1.499, 0.040 | 0.063 |
| ARDS | −0.208 | −1.001, 0.585 | 0.602 |
| Hemoglobin | −0.045 | −0.092, 0.002 | 0.060 |
| PaO₂ | 0.000 | −0.002, 0.001 | 0.482 |
| Donor age | −0.002 | −0.016, 0.011 | 0.760 |
| PGD grade 3 | −0.030 | −0.473, 0.412 | 0.891 |
| Ischemic time (h) | 0.015 | −0.035, 0.065 | 0.545 |
| OR time (h) | 0.051 | −0.057, 0.159 | 0.349 |
| ECMO use | −0.269 | −0.785, 0.247 | 0.300 |
| Time × Mixed–AMR | 0.045 | −0.100, 0.190 | 0.544 |

The -0.422 estimate for the “Mixed-AMR” variable indicates that the AMR-subtype is contributing more than the ACR-subtype towards the reduced post-transplant baseline FEV_1_ among patients with a mixed sensitization phenotype compared to the non-sensitized group (p=0.022). Interaction terms are included to study time-dependent changes in lung function across phenotypes. Adjusted β estimates for FEV_1_ baseline and trajectory are included in the table. BOLT, bilateral orthotopic lung transplantation; ILD, interstitial lung disease; COPD, chronic obstructive pulmonary disease; PAH, pulmonary arterial hypertension; ARDS, acute respiratory distress syndrome; PGD, primary graft dysfunction; ECMO, extracorporeal membrane oxygenation. Intercept represents modelled baseline lung function, measured by FEV_1_. ECMO use includes any ECMO use before, during, and after lung transplantation. Adjusted FEV_1_ trajectories are depicted in the table. Patients with at least two post-transplant FEV_1_ measurements were included in this analysis.

**Table S10**. Multivariable logistic regression for CLAD risk comparing ACR vs AMR-driven mixed sensitization.

| Variable | OR | 95% CI | p-value |
| --- | --- | --- | --- |
| **Mixed–AMR (reference: Mixed–ACR)** | 2.34 | 0.60–10.37 | 0.232 |
| Age (y) | 1.01 | 0.95–1.08 | 0.745 |

The odds ratio (OR) of 2.34 for the “Mixed-AMR” variable is non-significant (p = 0.232), but it may be suggestive that the AMR-subtype is the driver behind the trend observed in Table 4 that shows the “Mixed” sensitization with an OR = 2.36 (p = 0.060) compared to the non-sensitized group. This multivariable logistic regression only adjusts for age due to the limited number of CLAD events within the mixed sensitization group.

**Table S11**. Multivariable Cox proportional hazards model for overall survival comparing ACR vs AMR-driven mixed sensitization.

| Variable | HR | 95% CI | p-value |
| --- | --- | --- | --- |
| **Mixed–AMR (reference: Mixed–ACR)** | 2.17 | 0.92–5.14 | 0.078 |
| Age (y) | 1.00 | 0.97–1.04 | 0.974 |
| PGD Grade 3 | 1.83 | 0.66–5.09 | 0.245 |
| OR time (h) | 1.31 | 1.06–1.61 | 0.011 |

The hazard ratio (HR) of 2.17 for the “Mixed-AMR” variable is non-significant (p = 0.078), but it highlights a trend that the AMR-subtype is contributing more than the ACR-subtype towards the lower survival associated with a mixed sensitization compared to the non-sensitized group. This multivariable logistic regression only adjusts for age, PGD grade 3 status, and operating room time due to the limited number of death events within the mixed sensitization group.

**Table S12**. Patient counts in each phenotype for the 6-month landmark sensitivity analysis.

| Phenotype | Count (%) |
| --- | --- |
| Non-sensitized | 309 (73.6) |
| Pre-transplant humoral | 51 (12.1) |
| ACR sensitization | 43 (10.2) |
| Mixed sensitization | 17 (4) |
